# Novel approaches for left atrial pressure relief: Device-based monitoring and management in heart failure

**DOI:** 10.3389/fcvm.2022.910957

**Published:** 2022-08-11

**Authors:** Chihiro Miyagi, Taiyo Kuroda, Jamshid H. Karimov, Kiyotaka Fukamachi

**Affiliations:** ^1^Department of Biomedical Engineering, Cleveland Clinic, Lerner Research Institute, Cleveland, OH, United States; ^2^Cleveland Clinic Lerner College of Medicine of Case Western Reserve University, Cleveland, OH, United States

**Keywords:** device-based treatment, mechanical circulatory support, left ventricular assist device (LVAD), interatrial shunt device, diastolic dysfunction (DD), heart failure with preserved ejection fraction (HFpEF), left atrial monitoring, left atrial assist

## Abstract

The importance of the left atrium (LA) has been emphasized in recent years as the features of heart failure (HF), especially with regard to variability in patient and pathology phenotypes, continue to be uncovered. Of note, among the population with HF with preserved ejection fraction (HFpEF), pressure or size of the LA have become a target for advanced monitoring and a therapeutic approach. In the case of diastolic dysfunction or pulmonary hypertension, which are often observed in patients with HFpEF, a conventional approach with clinical symptoms and physical signs of decompensation turned out to have a poor correlation with LA pressure. Therefore, to optimize HF treatment for these populations, several devices that are applied directly to the LA have been developed. First, two LA pressure (LAP) sensors (Heart POD and V-LAP Device) were developed and may enable patient self-management remotely with LAP-guided and physician-directed style. Second, there are device-based approaches that aim to decompress the LA directly. These include: (1) interatrial shunt devices; (2) left ventricular assist devices with LA cannulation; and (3) the left atrial assist device. While these novel device-based therapies are not yet commercially available, there is expected to be a rise in the proposition and adoption of a wider range of choices for monitoring or treating LA using device-based options, based on LA dimensional reduction and optimization of the clinically significant pressure relief. Further development and evaluation are necessary to establish a more favorable management strategy for HF.

## Introduction

In the treatment of chronic heart failure (HF), left atrial (LA) function has been identified as one of the most important parameters affecting the quality of life and potential deterioration or improvement of left atrial unloading. In ~90% of hospitalizations for exacerbation of HF, there is pulmonary congestion related to an increase in LA pressure (LAP) (1–3). In daily medical care, however, current management strategies for ambulatory HF patients generally rely on clinical symptoms and physical signs of decompensation, even though these indicators have a poor correlation with LAP (4).

For patients with HF with preserved ejection fraction (HFpEF) or pulmonary hypertension, diastolic dysfunction or right HF are the main pathophysiological elements responsible for the clinical representation and overall course of the disease. These types of HF tend to be resistant to simple volume reduction or vasodilators, since these approaches do not directly reduce the LAP in case left ventricular (LV) systolic function is preserved. Even though patients with HFpEF account for nearly half of the entire HF population (5), there is still an unmet need for effective therapeutic options.

To achieve more dedicated HF control for this population, it is essential to understand the specific clinical requirements in order to have effective options for monitoring LAP, and accurately adjusted, effective LA decompression. In this mini-review, we describe several novel devices that are currently in the development pipeline to treat HF that specifically address the LA function and parameters. The features, advantages and limitations of these device platforms are discussed.

## Left atrial pressure sensors

Changes in volume status or ventricular function, followed by decompensation and severe symptoms in HF patients, often require hospitalization and invasive monitoring of the patient's clinical status to achieve optimal medication and volume control. To limit the number of hospitalizations, strong efforts have been made for several decades to develop an accurate and remote monitoring system for early detection of exacerbation of chronic HF conditions (6). The unprecedented era of the COVID-19 pandemic has emphasized the necessity of reliable remote monitoring of HF patients more than ever.

Currently, continuous and remote monitoring of pulmonary artery pressure (PAP) have been confirmed to be associated with a reduced number of hospitalizations and mortality rates in the HF with reduced ejection fraction (HFrEF) population (7, 8). Also, PAP-guided therapies (PAP sensors) are the only commercially available option for CHF management so far. However, PAP does not always reflect left-sided ventricular filling pressures (9, 10) as seen in advanced HF patients with increased pulmonary vascular resistance or patients with pulmonary hypertension or acute HF.

Under these circumstances, LAP direct monitoring systems (intra-cardiac pressure readings) were developed to provide more sensitive and important information, including the evaluation of diastolic function and atrial arrhythmias.

### Heart POD device

**Figure 1:**
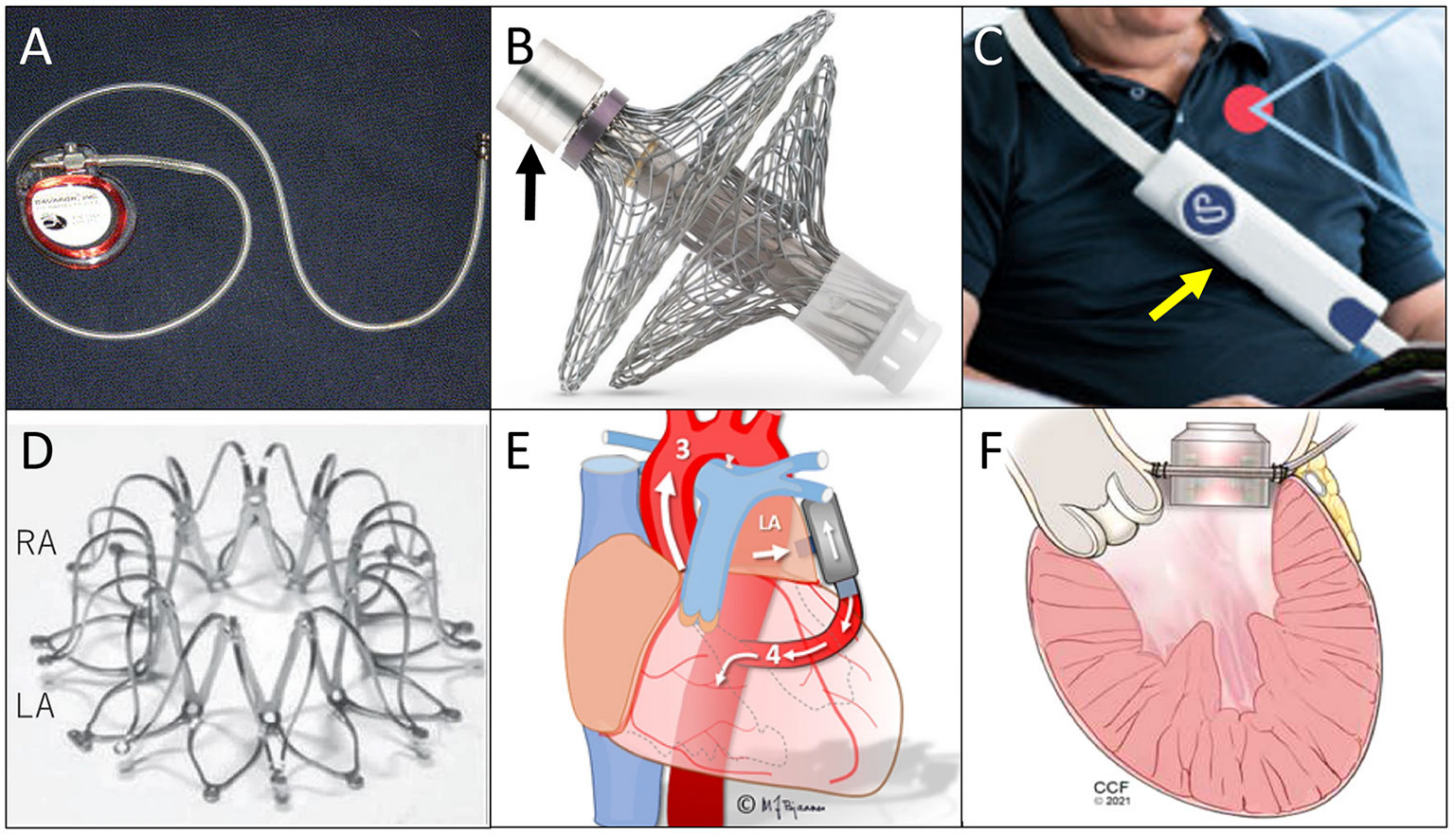
Illustrations of each device. **(A)** Heart POD, quoted from (6); **(B)** Sensory implant of V-LAP System (black arrow is the pressure-sensing assembly connected to electronic circuitry), quoted from (6); **(C)** Sensor of V-LAP (yellow arrow), quoted from (15); **(D)** Corvia Atrial Shunt Device, quoted from (19); **(E)** PulseVAD, quoted from (30), and; **(F)** Left Atrial Assist Device, quoted from (32).

The Heart POD (Abbott, Abbott Park, IL) was developed as the first attempt to place in a human a permanently implantable direct LAP monitoring device (11). This is a pacemaker-shaped device with a coil antenna implanted in the subcutaneous pocket and a sensor lead placed across the atrial septum (Figure 1A). A patient advisor module is used to communicate with the implanted sensor lead. In a prospective, multicenter, non-randomized, open-label feasibility clinical trial [the Hemodynamically Guided Home Self-Therapy in Severe Heart Failure Patients (HOMEOSTASIS) trial], the Heart POD was implanted in eight patients with established HF (11). At 12 weeks of follow-up, the device measurements were as accurate as within ±5 mm Hg of simultaneous pulmonary capillary wedge pressure readings, and no complications were reported.

As a follow-up to the HOMEOSTASIS trial, a prospective and observational study of a physician-directed patient self-management system targeting LAP was conducted, enrolling 40 patients with HFrEF and HFpEF and a history of acute decompensation (12). During pressure-guided therapy, mean daily LAP fell in the first 3 months from 17.6 to 14.8 mm Hg (*p* = *0.003*), and the frequency of LAP elevation higher than 25 mm Hg was reduced by 67% (*p*< *0.001*). In addition, improvements in New York Heart Association (NYHA) functional Class, LV ejection fraction, and pharmacological profiles were observed.

Following this, the Left Atrial Pressure Monitoring to Optimize Heart Failure Therapy (LAPTOP-HF) study was initiated (13). The LAPTOP-HF was a prospective, multicenter, randomized, controlled clinical trial, which was designed to enroll up to 730 patients with NYHA functional class II and either of a history of hospitalization for HF in past 12 months or an elevated B-type natriuretic peptide level, regardless of the LV ejection fraction. The enrollment was terminated early, however, because of a large number of procedure-related complications by trans-septal punctures. The analysis of 486 patients that were enrolled prior to the termination showed that the HF therapy, with LAP-guided, physician-directed, and patient self-management, was associated with a 41% reduction in HF hospitalizations at 12 months (*p* = *0.005*) (14). These efforts were followed by the development of the V-LAP, a more advanced LAP sensor.

### V-LAP remote monitoring system

The V-LAP System (Vectorious Medical Technologies, Tel Aviv, Israel), is a wireless remote monitoring system that measures LAP directly (2, 15). The system includes a sensory implant (Figure 1B) placed at the interatrial septum and an external unit (reader, Figure 1C). The implant is leadless, has no battery, and receives all its power from the reader. All procedures can be done percutaneously. A hermetically sealed tube encases the sensing elements and electronics, and bidirectional communications with a reader is enabled.

After *ex vivo* and *in vivo* animal experiences (16), the V-LAP Left Atrium Monitoring system for Patients With Chronic sysTOlic & Diastolic Congestive heart Failure (VECTOR-HF) study (ClinicalTrials.gov: NCT3775161) was recently initiated. This is a prospective, multicenter, single-arm, and open-label, first-in-human clinical study that aims to assess the safety, performance, and usability of the device in patients with NYHA class III HF. So far, 24 patients have received the device implants, which are transmitting accurate pressure measurements of LAP with no device-related complications or sensor failure events (2).

In general, the most serious concern with these LAP sensors is a higher rate of procedure-related complication. The V-LAP system seems to have less risk of thrombosis than the Heart POD because of its shape, but information about long-term biocompatibility and effectiveness are still needed.

## Depressurization of the left atrium

Decompression of the LA is the most ideal therapeutic approach to relief the symptoms and vicious circle of exacerbated HF. Especially in cases of HF with diastolic dysfunction, as represented by patients with HFpEF, pharmacological treatments have not been as feasible as they have been for HFrEF. Use of LV assist devices (LVADs) is also controversial because of limited experience and concerns over the risk of ventricular suction events. Although sodium glucose cotransporter 2 (SGLT2) inhibitors were recently suggested to be beneficial for HFpEF (17, 18), their efficacy is still unclear after fluid overload is appropriately managed. SGLT2 inhibitors brought a lower risk of hospitalization for HF, but there was no improvement in death rates. Therefore, new device-based therapies that address LA decompression have been getting more attention in the area of HFpEF treatment (19, 20).

There are three options for decompressing the LA with a device: (1) fenestrate the interatrial wall; (2) use LVADs with LA cannulation; and (3) pump blood directly from the LA to the LV. High LAP and large LA size are two of the most important features of HFpEF pathology, and reducing LA size and pressure have been set as important therapeutic targets (21, 22). Here, we summarize recent findings for each type of device.

### Interatrial shunt devices

Interatrial shunt devices are the most widely applied option for the HFpEF population. There are three different devices, used as artificial interatrial shunts (23): the Corvia Atrial Shunt Device (IASD System II, Corvia Medical Inc., Tewksbury, MA), the V-Wave device (V-Wave Ltd., Caesarea, Israel), and the Atrial Flow Regulator (AFR, Occlutech, Helsingborg, Sweden). They employ the same concept of creating a shunt between the LA and the right atrium (RA) and reducing LAP by generating left-to-right flow artificially. Their materials and shapes vary, but typically, a 5-to-10 mm shunt is made and fixed at the interatrial wall by a self-expanding prosthesis, and all procedures can be done percutaneously.

Among them, the Corvia Atrial Shunt Device (Figure 1D) has already undergone several randomized clinical trials. A randomized, multicentre (international), blinded, sham-controlled trial (REDUCE LAP-HF II) (24), enrolled 1,072 participants; 314 were assigned to the device-implanted group. The placement of an atrial shunt did not reduce the total HF event rates at 12 or 24 months after implantation. The authors stated that the strategy of excluding pulmonary vascular disease might have not been adequate. Thus, with better patient selection, atrial shunt devices still have a chance to be beneficial. Nevertheless, as an HF treatment, the efficacy of this device is limited to symptom relief, and it can never be used as a causal treatment nor to stop progression of the disease.

### Left atrial cannulation of left ventricular assist devices

As noted, LVADs are not considered to be as beneficial for patients with HF with diastolic dysfunction as for those with systolic dysfunction, because the LV wall in diastolic dysfunction is often too thick and the LV cavity too narrow for the LVAD inflow cannulas. The concept of applying LVADs with LA cannulation emerged, and some case reports depicted the potential efficacy of LVAD implantation in hearts with diastolic dysfunction, such as hypertrophic cardiomyopathy (25–27).

There are some novel devices that employ a similar concept of drawing blood from the LA and returning it to the aorta or subclavian artery. For example, the CircuLite Synergy Micro-Pump Device (Medtronic, Minneapolis, MN) (28) is a micropump-based form of mechanical circulatory support device, with a pump the size of an AA battery (29). The implantation can be done with a right mini-thoracotomy without using cardiopulmonary bypass, and the outflow graft is anastomosed to the subclavian artery. Although the development of the Synergy Micro-Pump Device and related project had been abandoned several years ago, this concept was successfully migrated to the new pumps, such as the VADovations cardiac assist system (VADovations, Oklahoma City, OK) or the PulseVAD (Northern Development, Strandhaugen, Oslo).

These two devices are at the beginning of the development process, and very limited information is available. Briefly, VADovations is a small pump the size of a AAA battery and is placed between the LA and the ascending aorta without inflow/outflow cannulas. The experimental results have not yet been documented. The PulseVAD is a pulsatile heart assist device and pumps blood from the LA to the descending aorta with minimally invasive surgery without cardiopulmonary bypass (Figure 1E). Gude and Fiane recently published the results of *in vivo* studies with the PulseVAD using ovine, and reported survival for 11 days after implantation without any complication (30).

Draining blood directly from the LA seems to be the most reasonable method of decreasing LAP, but these devices make an alternative bloodstream by shortcutting the LV. Even if they are intended as partial circulatory support, stagnation in the LV and a risk of LV thrombosis are inevitable. Also, pulsatility should be decreased as the pump support becomes larger.

### Left atrial assist device

Sharing the concept of draining blood from the LA, the Left Atrial Assist Device (LAAD) has a unique feature of being implanted at the mitral position and pumping blood directly to the LV (Figure 1F) (31). The target of this pump is mainly HFpEF or diastolic dysfunction with normal EF, since the systolic function of the heart needs to be maintained for the native LV to pump blood by itself. For use in hearts with preserved EF, the LAAD can decrease LAP and support the LV filling, maintaining physiological blood pathway and pulsatility.

**Figure 2:**
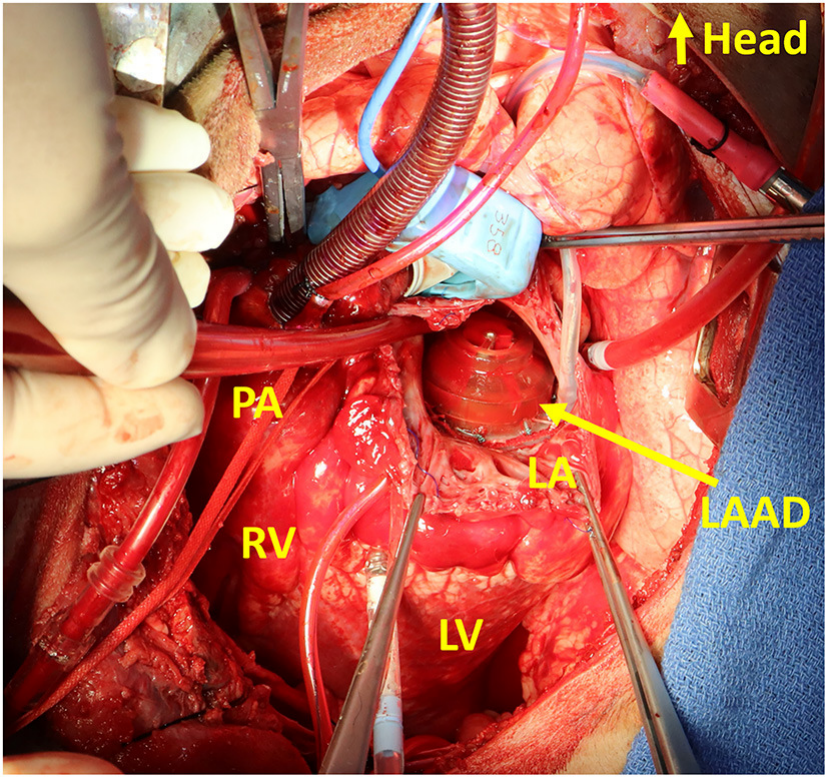
Intraoperative photo of the Left Atrial Assist Device (LAAD) implanted at the mitral position. LA, left atrium; LV, left ventricle; PA, pulmonary artery; RV, right ventricle.

Our progress with the LAAD was reported with *in vitro* and *in vivo* studies with calves (32, 33). The intraoperative image of the LAAD implanted at the mitral position is seen as Figure 2. The effect of reducing LAP has been successfully demonstrated with introduced diastolic HF models. The work on reduction of the device profile, which would allow shrinking of the device dimensions and favor the implantability and technology footprint inside the heart, is ongoing. Driveline exteriorization strategies are pending evaluation, and would provide more information on the most appropriate anatomical considerations and surgical options for its pass through cardiac structures. The evaluation with long-term safety and efficacy is needed, as well as the development of animal models that can simulate diastolic HF with more fidelity.

## Discussion

The importance of LA function has been gradually and steadily emphasized as the concept of diastolic dysfunction has become more popular in the HF field. Decreased LA compliance and mechanics are believed to be associated with an increased risk for new onset atrial fibrillation in HFpEF (34), and reducing the LA size and pressure has become the key treatment strategy. However, considering LA as a therapeutic target requires tremendous effort, since obtaining the precise value of LAP requires invasive catheterization in the hospital.

Whether performed percutaneously or surgically, there is an inevitable risk of systemic thrombosis when any prosthetic is introduced into the LA, and so anticoagulation and/or antiplatelet therapies are usually prescribed (6, 35). Therefore, for these LA devices, patient selection is very important. Patients with diastolic dysfunction as represented by HFpEF or right HF, including HF with pulmonary hypertension, would be good candidates, since LA function plays larger roles.

The devices introduced here are all in development, and none has obtained approval from the U.S. Food and Drug Administration (FDA). Their effects on early detection or prevention of LA arrhythmia have not been demonstrated, and it's also unclear if they have any therapeutic effect on presenting arrhythmia. However, under the social, medical, and economic crises brought on by the COVID-19 pandemic, a case was reported in which constant tele-monitoring of LAP and subsequent adjustment of medication prevented possible decompensation of HF and hospitalization in a patient who was in self-isolation (15). With more evidence and the need for remote care, continuous remote monitoring by invasive sensors is expected to play a larger role in HF care.

As for the LA decompression devices, the Corvia Atrial Shunt Device received FDA breakthrough device designation in 2019, but it failed to show a long-term efficacy in a randomized trial. The pump-based devices should have promise for decompressing LA, but their development is still at the animal experiment stage, and needs more time before a first-in-human trial. Also, pump devices-implantation tends to be more invasive.

## Conclusion

With the new device-based options, there is a wider range of choices for monitoring or treating LAP. Further development and evaluation are required to establish a more favorable management strategy for HF.

## Author contributions

CM performed the literature search, designed the study, and prepared the manuscript. KF, JK, and TK provided methodological support in the study design and revised the manuscript. All authors have read and approved the manuscript before submission.

## Funding

The LAAD study was supported by funding from National Heart, Lung and Blood Institute, National Institutes of Health (NIH), NIH Center for Accelerated Innovation at Cleveland Clinic (NCAI-CC), (NIH-NHLBI 1UH54HL119810; NCAI-19-12-APP-CCF).

## Conflict of interest

Authors KF and JK are co-inventors of the LAAD. The remaining authors declare that the research was conducted in the absence of any commercial or financial relationships that could be construed as a potential conflict of interest.

## Publisher's note

All claims expressed in this article are solely those of the authors and do not necessarily represent those of their affiliated organizations, or those of the publisher, the editors and the reviewers. Any product that may be evaluated in this article, or claim that may be made by its manufacturer, is not guaranteed or endorsed by the publisher.
